# A validated restriction enzyme ddPCR cg05575921 (*AHRR*) assay to accurately assess smoking exposure

**DOI:** 10.1186/s13148-024-01659-1

**Published:** 2024-03-25

**Authors:** Sandra Fitzgerald, Basharat Bhat, Cristin Print, Gregory T. Jones

**Affiliations:** 1 Department of Molecular Medicine & Pathology, The University of Auckland, Auckland, New Zealand; 2 Maurice Wilkins Centre of Research Excellence, Auckland, New Zealand; 3 Vascular Research Group, Department of Surgical Sciences, Dunedin Medical School, University of Otago, PO Box 56, Dunedin, 9054 New Zealand

**Keywords:** Aryl hydrocarbon receptor repressor (AHRR), Bisulfite conversion, DNA methylation, Droplet digital PCR, Epigenetics, Restriction enzyme, Smoking exposure

## Abstract

**Background & Methods:**

In this study, a novel restriction enzyme (RE) digestion-based droplet digital polymerase chain reaction (ddPCR) assay was designed for cg005575921 within the *AHRR* gene body and compared with matching results obtained by bisulfite conversion (BIS) ddPCR and Illumina DNA methylation array.

**Results:**

The RE ddPCR cg05575921 assay appeared concordant with BIS ddPCR (*r*^2^ = 0.94, *P* < 0.0001) and, when compared with the Illumina array, had significantly better smoking status classification performance for current versus never smoked (AUC 0.96 versus 0.93, *P* < 0.04) and current versus ex-smoker (AUC 0.88 versus 0.83, *P* < 0.04) comparisons.

**Conclusions:**

The RE ddPCR cg05575921 assay accurately predicts smoking status and could be a useful component of ‘precision-medicine’ chronic disease risk screening tools.

**Supplementary Information:**

The online version contains supplementary material available at 10.1186/s13148-024-01659-1.

## Introduction

Numerous epigenome-wide association studies have reported robust and reproducible DNA methylation changes correlated with smoking exposure, with the strongest association consistently being cg05575921, within the gene body of *AHRR* [1, 2]. Sensitive PCR methods have subsequently been developed to assess the methylation status of cg05575921 [3–5]. These techniques typically utilize bisulfite conversion, which is a harsh chemical process resulting in significant DNA degradation and can be associated with issues regarding conversion efficiency if not carefully conducted [6]. Alternative PCR assays have been reported which avoid the need for bisulfite conversion by utilizing methylation-sensitive restriction enzyme digestion. Unfortunately, the pattern of methylation levels generated does not always appear to closely match those typically associated with bisulfite conversion and DNA methylation array-based measures of this specific CpG site [7]. In this study, we aimed to develop a restriction enzyme digestion-based droplet digital PCR (ddPCR) assay for cg05575921 (*AHRR*) that produces comparable results with that of bisulfite conversion-based methods. We further confirm its sensitive and specific association with smoking status in a large clinically relevant elderly cohort.

## Methods

This observational cohort study examined a subset of participants recruited as part of the New Zealand Vascular Genetics Research Programme. In brief, consecutively recruited participants of an abdominal aortic aneurysm screening project and a healthy age matched control group were examined [8]. Each participant was over 50 years of age and had self-reported smoking status (never, ex-smoker, current smoker), smoking pack years, age and biological sex data collected at time of recruitment. One pack year was defined as twenty cigarettes (or tobacco equivalent) smoked every day for one year and was calculated using the publicly available online tool https://www.smokingpackyears.com.

For the initial ddPCR setup and validation experiments (Additional file 1: Sheets S1 and S4), separate subgroups of never, ex and current smokers (with high pack year consumption) were purposefully selected (without age or sex matching) selected, but sample status was blinded during ddPCR assessment.

A detailed description of the DNA extraction and the two droplet digital PCR methods utilized is provided in Additional file 2. In brief, genomic DNA was extracted from EDTA-anticoagulated whole blood using a modified salting-out method and stored at − 20 °C until analysis. DNA concentration was determined using a nanophotomer (Implen P360).

A custom ddPCR assay was designed to quantify the proportion of methylated copies of cg05575921. This assay utilized the methylation-sensitive nature of the restriction enzyme HpaII (RE ddPCR). HpaII digestion was performed on purified gDNA and the digested DNA used as template for the subsequent ddPCR. Quantification of the number of methylated cg05575921 CpG sites was performed using a FAM-labeled probe. To allow for normalization of the RE ddPCR assay, a HEX-labeled probe was designed to a region of the gene *KIT* that did not contain any HpaII cut sites. The per cent methylation of the cg05575921 *AHRR* CpG site was determined by dividing FAM (methylated cg05575921) by HEX (copies of *KIT*), each being calculated by Poisson distribution (cp/µL) according to manufacturer’s guidelines.

A second ddPCR assay, based on the bisulfite (BIS) ddPCR assay published by Arroyo et al. [3], was used for comparison with the RE ddPCR approach. The primers and probes were as previously published [3] with the exception of the forward primer which was redesigned in Primer3Plus [9] to achieve a smaller amplification product of 126bp. Neither primers nor probes contained polymorphic bases with global population minor allele frequencies greater than 0.1%. Bisulfite-converted (EZ DNA Methylation Kit (Zymo)) gDNA was used as template for subsequent ddPCR. In this assay, cg05575921 methylation was assessed using a methylated-site-specific FAM probe and an unmethylated-site-specific HEX probe. Because this assay assessed FAM and HEX signal, assessing the same amplicon quantification was performed using fraction abundance (per cent methylated = FAM divided by the total of FAM and HEX, each probe being measured as cp/µL).

Illumina Infinium MethylationEPIC (v1) array data were generated as previously reported [8]. Raw idat files were processed using the GenomeStudio software (Illumina) and Bioconductor packages ChAMP [10] and minifi (v1.46) [11]. Several different iterations of normalization schema were applied to the array data, including Noob, SWAN, BMIQ and GenomeStudio [12]. Normalized beta-values for cg05575921 were then extracted for paired comparisons to ddPCR results generated using matching DNA samples. Results for all techniques were reported as a percentage of methylation for ease of comparison.

Statistical analysis was conducted by assessing paired correlations between assays using Fischer Z-transformation of the Pearson correlation coefficient. The agreement between assays was tested using Bland–Altman analysis. The smoking status classification performance of the assays was assessed by receiver operating characteristic (ROC) curve analysis, with the method of Delong et al. [13] being used to calculate of the standard error of the area-under-the-curve (AUC). Pairwise comparisons were conducted between the respective AUC generated by the EPIC array and RE ddPCR assays. Optimized smoking status group association thresholds were calculated using the Youden J statistic and tabulated with their corresponding sensitivity and specificity measures. Confusion matrices were used to present the positive and negative predictive performance of the dichotomized threshold values. Statistical tests were performed using MedCalc version 19.2 (MedCalc Software, Ostend, Belgium).﻿

## Results

The overall cohort examined in this study (*n* = 1227, Table 1) had a mean age of 70 years, 71% were male, and 37, 51 and 12% were never, ex or current smokers, respectively.

**Table 1:** Demographics of cohort comparing ddPCR and EPIC array cg05575921 values

	Total	Never smoked	Ex-smoker	Current smoker
Number	1227	450	630	147
Sex, *n* of males (%)	871 (71.0)	300 (66.7)	473 (75.1)	98 (66.9)
Age (years)	70.1 ± 8.2	70.8 ± 7.6	70.6 ± 8.2	65.6 ± 8.9
Smoking pack years	7 (0–30)	0	20 (8–38)	34 (21–48)
History of cardiovascular disease*	708 (57.7)	214 (47.6)	367 (58.3)	127 (86.4)
cg05575921, Illumina array % DNA methylation	78.3 ± 12.2	84.7 ± 7.3	77.6 ± 10.8	61.4 ± 12.8
cg05575921, RE ddPCR % DNA methylation	75.8 ± 16.2*	84.7 ± 8.3	75.4 ± 14.2*	50.5 ± 15.3*

Restriction enzyme (RE) digestion ddPCR cg05575921 (*AHRR*) values were significantly lower compared to array derived values (*paired *t* test, *P* < 0.0001) in both ex and current smokers. There was no significant difference (*p* = 0.28) between values in never smokers. Cardiovascular disease included documented medical history of ischemic stroke or transient ischemic attack, coronary heart disease or peripheral artery disease [8]. Continuous measures are represented as either mean ± 1 standard deviation or median and interquartile range

It has been previously demonstrated that a bisulfite-converted DNA ddPCR assay can accurately determine the CpG methylation status of cg05575921 within *AHRR* [3, 4, 14]. Furthermore, strong concordance between BIS ddPCR and Illumina Epic array measures of cg05575921 has been reported [4, 14]. In this study we wanted to investigate whether a restriction digestion approach coupled with droplet digital PCR could also be used to accurately measure the level of methylation of the cg05575921 CpG site. Initial investigations were conducted on gDNA isolated from 52 cohort participants (23 current smokers, 6 ex-smokers and 23 never smokers) which were assessed using both the cg05575921 RE and BIS ddPCR assays. There was a strong correlation between measures (*r*^2^ = 0.94, *P* < 0.0001), with an overall coefficient of variance between the two assay types of 5.2% (Additional file 1: Sheet S1). To investigate the quantitative consistency of the restriction enzyme approach, the RE ddPCR assay was repeated for 16 separate samples (2–6 replicates), as well as a technical replicate included in each 96-well batch. The RE ddPCR assay had good technical reproducibility in the 16 individual replicates, with a mean coefficient of variation of 6.0% (Additional file 1: Sheet S1). The plate batch replicate sample had a coefficient of variation of 3.2% (mean methylation 91.4 ± 2.9%, *n* = 12 batch replicates).

Because the RE ddPCR assay involved the amplification of two separate PCR products (*AHRR* and *KIT* amplicons), we performed an additional analysis to confirm that there was no PCR amplification bias between the two primer/probes sets. This involved comparison of FAM and HEX copies/µL in ddPCR reactions which had not undergone restriction enzyme digest. (as described in the supplementary methods, with results in Additional file 1: Sheet S4). There was no strong bias in amplification between the two PCR reactions, and therefore, inclusion of no enzyme normalization made no significant difference to the smoking status predictive performance of the assay Additional file 1: Sheet S4 Table 2).

We then examined the effect of different normalization schema on Illumina Infinium MethylationEPIC (v1) array derived cg05575921 values. Although there did appear to be a subtle influence on cg05575921 values across smoking status groups (Additional file 1: Sheet S2), this did not significantly (all pairwise ROC-curve comparisons *P* > 0.05) influence the smoking status classification performance of array derived values and, given this equipoise and its wide use in the literature, subsequent results are reported using Illumina Genome Studio normalization.

Next, we examined the full set of 1227 samples, matching cg05575921 RE ddPCR results with their respective Illumina EPIC array data. The comparative quantitation of DNA methylation at cg05575921 is shown in Table 1 and Additional file 1: Sheet S3.

Within the never smoked group, there was no statistically significant difference (*p* = 0.28) between RE ddPCR and Illumina array cg05575921 methylation values. However, the RE ddPCR values were significantly lower compared to their matching Illumina array values in both the ex (*t*-value 4.7, *P* < 0.0001) and current (*t*-value 8.5, *P* < 0.0001) smoker groups, with this difference being most apparent in current smokers (Table 1 and Additional file 1: Sheet S3B).

Bland–Altman analysis also demonstrated that disagreement between paired test measures was not uniform (Additional file 1: Sheet S3C), with good agreement in values greater than 80% methylation (the majority of whom were never smokers), but increasingly poorer agreement within values 65–80% (mostly ex-smokers) and less than 65% (mostly current smokers). This was also evident in the nonlinear correlation between measures (Additional file 1: Sheet S3D).

**Fig. 1:**
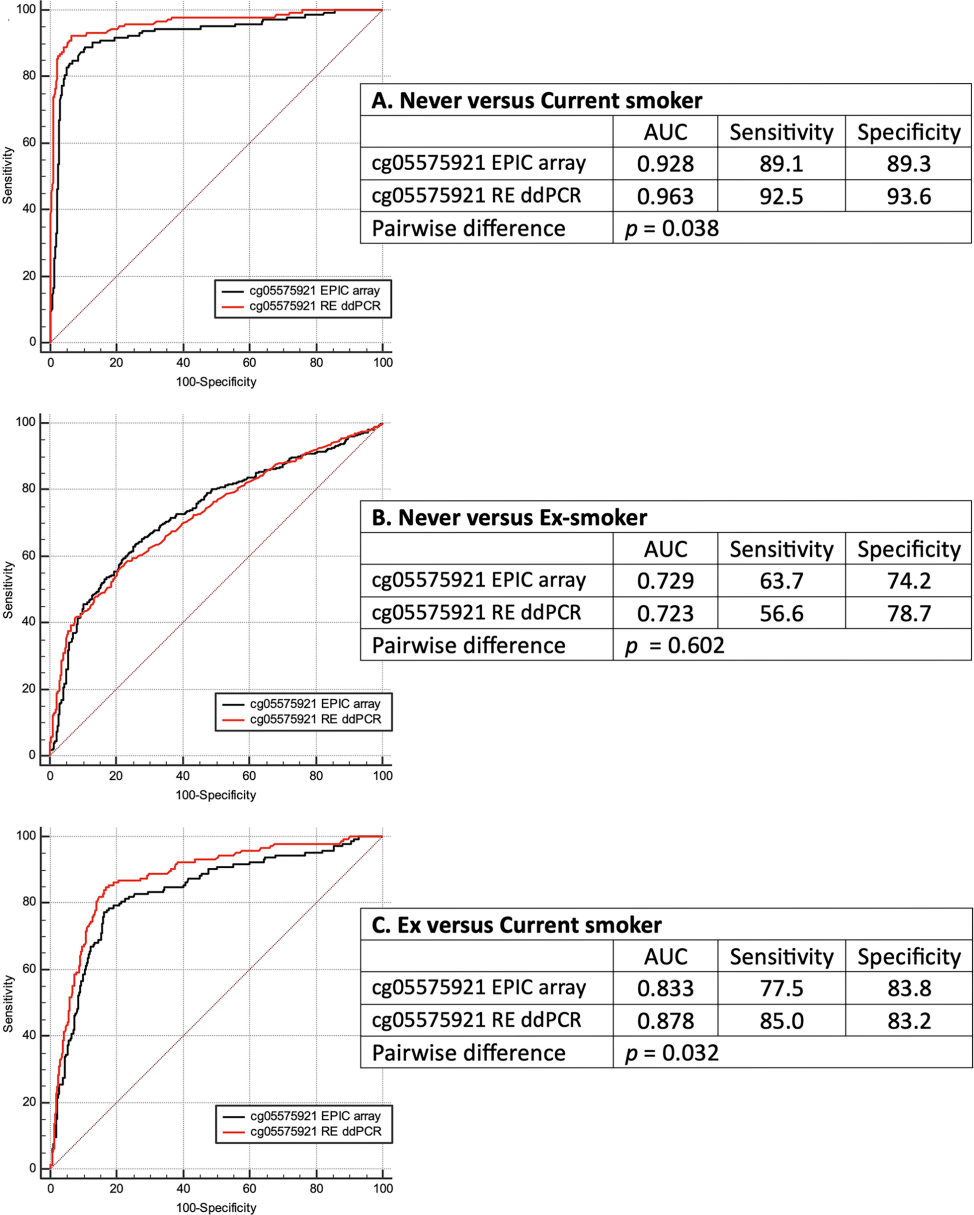
Relative sensitivity and specificity of cg05575921 assays detection of smoking status. DNA methylation values for cg05575921 derived from Illumina EPIC assays (GS normalised) and RE ddPCR were significantly predictive of smoking status (all AUCs *P* < 0.0001), however, the RE ddPCR assay was significantly more sensitive and specific in differentiating between **A** never and current smokers and **C** Ex and current smokers. **B** There was no significant difference in the predictive performance of either assay to distinguish between never and ex-smokers. Confusion matrices, tabulating the positive and negative predictive performances, are shown in Additional file 1: Sheet S3, Table 2.

Finally, the smoking status classification performance of cg05575921 RE ddPCR was compared with that of EPIC array using ROC-curve analysis. The RE ddPCR values had statistically better predictive performance, as assessed by pairwise differences in AUC, as well as positive and negative predictive values (confusion matrices in Additional file 1: Sheet S3 Table 2) for the current versus never smoked (Fig. 1A) and current versus ex-smoker (Fig. 1C) comparisons.

## Discussion

Smoking has long been recognized as a leading risk factor for a wide range of chronic conditions, including cardiovascular diseases and cancers [15]. Current clinical practice incorporates patient self-reported measures of smoking exposure, typically by assessing smoking status (never, ex or current smoker). Limitations and potential inaccuracies of such self-reported data have been suggested. For example, there is likely to be a wide range of smoking-related risk among ex-smokers depending on their volume of exposure (pack years) and duration of cessation. Similarly, second-hand exposure is not readily quantified with such measures.

Epigenetic markers, such as DNA methylation, are emerging as ‘precision medicine’ biomarkers that can surpass the performance of such ‘conventional’ markers in risk prediction [16]. This is particularly true for smoking, for which numerous epigenome-wide association studies have demonstrated robust and reproducible DNA methylation changes correlated with smoking exposure, with the strongest association consistently being cg05575921, within the gene body of *AHRR* [1, 2]. Consequently, sensitive PCR methods have been developed to assess the methylation status of this specific CpG site [5, 17]. The use of cg05575921 (*AHRR*) assays have been suggested to have the potential to improve risk prediction utility by a range of effects including assessment of second-hand smoke exposure [18], correcting for false or inaccurate self-reported measures [19] and by virtue of strong morbidity and mortality associations, even after adjusting for self-reported smoking history [5].

In this study, we report a technically accurate restriction enzyme digestion ddPCR assay, which appears to have similar performance to that of previously reported ddPCR assays [3, 4] without the need for the chemically harsh bisulfite conversion process [6], which can be an issue particularly when working with samples containing limited amounts of input DNA. However, previous methylation-sensitive restriction enzyme-based techniques do provide some reason for caution regarding this approach. Bravo-Gutierrez and colleagues reported a HpaII restriction enzyme digest method, but using qPCR rather than ddPCR as the detection platform. Unfortunately, the methylation patterns reported for the Bravo-Gutierrez assay do not match those typically associated with bisulfite conversion PCR and Illumina array-based measures of this specific CpG site [7]. Specifically, there was a substantial overlap in cg05575921 values when comparing smoking and non-smoking groups, and many current smokers were reported as having 100% methylation. This is contrary to the well-established literature, which consistently reports cg05575921 methylation values of 50–60% in the vast majority of current smokers [2, 17]. Nevertheless, other groups have convincingly demonstrated that some RE-based ddPCR assays can be highly sensitive and specific [20]. In addition, RE-based approaches are substantially less time-consuming than that of bisulfite conversion (30–60 min versus 12–16 h).

In contrast to the qPCR-based observations of the Bravo-Gutierrez assay, the RE ddPCR assay developed in this study produced results that are consistent with the prior literature [2, 5, 17]. Regardless of which of the three (RE or BIS ddPCR or Illumina array) assessing techniques used, we observed that the hypomethylation of the cg05575921 DNA methylation site was statistically significantly, sensitively and specifically associated with smoking status. Interestingly, using a large cohort of 1227 individuals, we were also able to confirm that the RE ddPCR assay had significantly better predictive power for smoking status than measures derived from Illumina arrays. We do, however, note a slight potential difference with a previous report by Philibert et al. [4], who showed a strong and linear (*R*^2^ = 0.98) correlation between EPIC array and BIS ddPCR cg05575921 results (in 92 sample comparisons) which did not match the nonlinear correlation in our current RE ddPCR versus EPIC array comparison (Additional file 1: Sheet S3). The quantification of the BIS and RE ddPCR differed, with fractional abundance assessment being utilized in the (single amplicon) BIS ddPCR assay and, in contrast, the RE ddPCR method involved comparison of two separate amplicons (*AHRR* and *KIT*), therefore necessitating comparison via a simpler ratio of FAM/ HEX signal. Nevertheless, we still observed a strong linear correlation between the two (BIS and RE) ddPCR methods, and the reason for the nonlinear effect observed between the RE ddPCR and EPIC array paired comparisons remains unclear. One possible explanation that we considered was the effect of array normalization. This is an essential step in the analysis of array data that is needed to reduce the size of batch effects, correct for probe type bias and adjust for background noise and dye bias [12, 21]. It is worth noting that ddPCR assays do not require such correction, as they are internally calibrated, in the case of the restriction enzyme assay to the number of copies of a reference gene (*KIT*). While we did observe subtle differences due to the normalization method that was applied to the array data (Additional file 1: Sheet S2), the lower RE ddPCR values observed in current smokers (Additional file 1: Sheet S3B) was a much larger effect and an explanation for this particular observation therefore remains elusive. Nevertheless, an apparent consequence of this ‘skewing’ of the hypomethylated signal appeared to be associated with a more sensitive and specific smoking group classification performance for the RE ddPCR assay compared to array derived measures. Indeed, as shown in Additional file 1: Sheet S3A, we noted several (10 of 147, 6.8%) known current smokers that, while having RE ddPCR values (below 60%) consistent with their smoking status, had Illumina array values (over 80%) which were more consistent with never smoked status.

It has also been suggested that normalization, using a separate no RE reaction, may be required for RE ddPCR assays [20]. In a sub-analysis, we demonstrated that inclusion of a no enzyme normalization step did not significantly alter the high smoking status sensitivity and specificity of this particular DNA methylation ddPCR assay (Additional file 1: Sheet S4).

In conclusion, we report the development of a restriction enzyme digestion-based droplet digital PCR assay for cg05575921 (*AHRR*) that produces at least comparable results to that of previously reported ddPCR and Illumina array-based methods without the need for bisulfite conversion of the DNA sample. We further confirm that this RE ddPCR method is able to sensitively and specifically predict smoking status in a large clinically relevant elderly cohort. This bisulfite conversion-free ddPCR assay appears to have potential clinical utility as a precise molecular diagnostic method for the evaluation of smoking exposure.

### Supplementary Information

**Additional file 1.** Supplementary file 2: Comparison of cg05575921 restriction enzyme versus bisulfite conversion ddPCR assays. Supplementary file 3: Effect of Illumina array normalisation on cg05575921 values. Supplementary file 4: Comparison of cg05575921restriction enzyme ddPCR versus Illumina EPIC array values. Supplementary file 5: Effect of inclusion of a no enzyme normalisation to the cg05575921 RE ddPCR assay.

**Additional file 2.** Supplementary file 1: ddPCR methods.

## Data Availability

Genome-wide datasets analyzed during the current study are not publicly available due to ethics approval restrictions. The ddPCR datasets analyzed during the current study are available from the corresponding author on reasonable request.
