# Supplementary material for: A validated restriction enzyme ddPCR cg05575921 (AHRR) assay to accurately assess smoking exposure
Source: Clin Epigenetics. 2024 Mar 25;16:45. doi: 10.1186/s13148-024-01659-1 (PMC10962207; doi:10.1186/s13148-024-01659-1)
Supplement: Supplementary file 2 — Additional file 2. Supplementary file 1: ddPCR methods. [file 13148_2024_1659_MOESM2_ESM.pdf]

Supplementary file 1

Separation of genomic DNA (gDNA)

For each patient sample, a (EDTA) blood pellet was generated by centrifugation for 15 minutes at 1500g at room temperature. After discarding the supernatant, the pellet was resuspended in 10mL of ice chilled 0.17M NH_4CL for 20 minutes. Following further centrifugation (as above) the resulting pellet was resuspended in 5mL PBS, before addition of 5mL of lysis buffer (20mM Tris pH 7.5, 50mM EDTA, 1% SDS), then addition of 2.5mL of 7.5M ammonium acetate (NH_4OAc). After centrifugation for 10 minutes at 1500g, the supernatant was added to 30mL of 100% alcohol to precipitate out the DNA as an aggregated flocculum. The DNA was removed using a clean glass rod and washed with 500µL 70% ethanol. The spooled gDNA left at room temperature for any remaining ethanol to evaporate before being resuspended in 500uL of low TE buffer (10mM Tris, pH 8.0, 0.1mM EDTA, pH 8.0).

Droplet digital PCR reactions

All ddPCR reactions were carried out on a Bio-Rad QX200 system, in a 23µL reaction with 1xddPCR Supermix for Probes (no dUTP) (Bio-Rad Laboratories, #1863025). Reaction template consisted of 5µL methylation sensitive restriction enzyme (RE) digested DNA or 3µL bisulfite converted DNA. These were mixed with HEX (209nm) and FAM (435nm) probes (each at 20µM), along with the target specific forward and reverse primers (10µM). Annealing temperature optimisation was carried out, and the following optimal amplification conditions were subsequently used: 1 denaturation cycle 95°C for 10 minutes, 40 cycles of 94°C for 30 seconds and 58°C for 1 minute (ramp rate 2°C per second) followed by a final 98°C for 10 minutes. Samples with >10,000 droplets generated were analysed using the QuantaSoft (version 1.0.596) software. The concentration of FAM and HEX probe target copies (cp) was calculated via Poisson distribution (cp/µL) according to manufacturer's protocol [2].

Restriction Enzyme (RE) ddPCR assay design

For the RE ddPCR assay, participant gDNA was digested with the methylation sensitive restriction enzyme HpaII. Unmethylated cg05575921 CpG sites will cleave, while methylated CpG sites will remain intact. Restriction digestion of the purified gDNA was performed on 1µL of gDNA (ranging from 60-150ng/µL). To each sample, 1µL of 10unit/µL FastDigest HpaII (ThemoFischer Scientific #FD0514) methylation sensitive restriction enzyme was combined with 3µL of 10x FastDigest Buffer (ThermoFisher Scientific) and each sample was adjusted to a final volume of 30µl in ultrapure nuclease free water. Samples were digested for 15 minutes at 37°C, and heat inactivated at 85°C for 5 minutes. Following digestion, 5µL of each sample was used as template for ddPCR, and the remaining digest was stored at -20°C.

The quantification of intact (methylated) DNA was determined with a FAM labelled ddPCR assay (with a 103bp amplicon). To allow for normalisation of the RE ddPCR assay, primers and a HEX probe was designed to a region of the gene *KIT* (a 102bp amplicon) that did not contain any HpaII restriction sites. The percent methylation of the cg05575921 *AHRR* CpG site was determined by dividing FAM (methylated cg05575921 cp/µL) by HEX (as a cp/µL measure of the total number of DNA (*KIT* gene) strand copies in the sample).

Restriction Enzyme (RE) ddPCR primer and Probe sequences	
Forward primer *AHRR (*RE_AHRR_F)	TGGGGACTGTTCACCTCTG
Reverse primer *AHRR (*RE_AHRR_R)	AATGAGAGGCTGGAAGGCT
FAM probe for *AHRR (*RE_AHRR_FAM)	/56-FAM/TGCACCCGG/ZEN/CTGGGTCTCA/3IABkFQ/
F primer for *KIT (*RE_KIT_F)	ACAGTGGAAGGTTGTTGAGG
R primer for *KIT (*RE_KIT_R)	AGCCTGTTTCTGGGAAACTC
HEX probe for *KIT (*RE_KIT_HEX)	/5HEX/ACATAGACC/ZEN/CAACACAACTTCCTT/3IABkFQ/

In addition, a sub-study was performed to determine the effect of normalising the RE ddPCR with a matching ddPCR reaction that did not contain the restriction enzyme (replaced by 1µL of ultrapure nuclease free water) as suggested by van de Leemkolk *et al* 2022 [2].

$$\text{Methylated cg05575921 (AHRR) fraction} = \frac{(\text{FAM/HEX})_{\text{RE ddPCR}}}{(\text{FAM/HEX})_{\text{No RE ddPCR}}}$$

FAM and HEX values as cp/µL (calculated via Poisson distribution)

This analysis suggested that, for this specific ddPCR assay, there is no significant difference in the resulting cp/µL of FAM versus HEX in undigested samples, indicating that there was no substantive amplification bias between the two (*AHRR* and *KIT*) PCR reactions. ROC-curve analysis confirmed that there was no significant difference in terms of smoking status prediction between values generated using (1) RE ddPCR or (2) RE ddPCR normalised using a no RE reference reaction (Suppl file 3).

Bisulfite ddPCR assay design

This assay was based on the bisulfite (BIS) ddPCR assay published by Arroyo et al 2022 [3]. The primers and probes were as previously reported [3], with the exception of the forward primer which was redesigned in Primer3Plus [4] to achieve a smaller amplification product of 126bp.

Bisulfite conversion of (300-600ng) gDNA was performed with the EZ DNA Methylation Kit (Zymo) as per the manufacturers protocol. Bisulfite converted samples were incubated for 15 hours at 50°C and held at 4°C for up to 8 hours before processing. The bisulfite converted DNA was eluted in 15µL of elution buffer and stored at -20°C. 3µL of bisulfite converted DNA was used as template for ddPCR.

For the bisulfite ddPCR assay, cg05575921 *AHRR* percent methylation was calculated using the Fractional Abundance method by dividing the concentration of FAM (methylated cg05575921 copies per µL) by the total of FAM and HEX (unmethylated cg05575921 copies per µL).

**Bisulfite ddPCR primer and Probe sequences**	
Forward primer *AHRR (*BIS_AHRR_F)	TGAGAGGGTAGTTTTGTTT
Reverse primer *AHRR (*BIS_AHRR_R)	AAACCCTACCAAAACCACT
FAM probe for *AHRR (*BIS_FAM)	/56-FAM/TTGTTTTGT/ZEN/ATTCGGTTGGGT/3IABkFQ/
HEX probe for *AHRR (*BIS_HEX)	/5HEX/TTGTTTTGT/ZEN/ATTTGGTTGGGTTT/3IABkFQ/

**PCR Reaction volumes**						
**BS ddPCR**		x 1		**RE ddPCR**		x1
	2 x MasterMix*	11.5			2 x MasterMix*	11.5
F primer	10 uM BIS_AHRR_F	1.44		F primer	10uM RE_AHRR_F	1
R primer	10uM BIS_AHRR_R	1.44		R primer	10uM RE_AHRR_R	1
Fam probe	20uM BIS_FAM	0.24		F primer	10uM RE_KIT_F	1
Hex probe	20uM BIS_HEX	0.24		R primer	10uM RE_KIT_R	1
	Water	5.14		Fam probe	20uM RE_AHRR_FAM	0.24
	DNA Template	3		Hex probe	20uM KIT_WT Hex	0.24
	**TOTAL**	**23µL**			Water	2.02
					DNA Template	5
					**TOTAL**	**23µL**

*1xddPCR Supermix for Probes (no dUTP) (Bio-Rad Laboratories, #1863025)

References

1. Basu AS. Digital Assays Part I: Partitioning Statistics and Digital PCR. SLAS Technol. 2017;22(4):369-86.
2. van de Leemkolk, FEM et al., Quantification of Unmethylated Insulin DNA Using Methylation Sensitive Restriction Enzyme Digital Polymerase Chain Reaction. Transpl Int, 2022. 35: 10167. https://doi.org/10.3389/ti.2022.10167
3. Arroyo, K., et al., Development of a Droplet Digital PCR DNA methylation detection and quantification assay of prenatal tobacco exposure. Biotechniques, 2022. 72(4): p. 121-133. PMID: 35255733 DOI: 10.2144/btn-2021-0099
4. Untergasser, A., et al., Primer3Plus, an enhanced web interface to Primer3. Nucleic Acids Res, 2007. 35(Web Server issue): p. W71-4. PMID: 17485472 DOI: 10.1093/nar/gkm306
